# Modulation of Human Complement System by Antimicrobial Peptide Arenicin-1 from *Arenicola marina*

**DOI:** 10.3390/md16120480

**Published:** 2018-12-01

**Authors:** Ekaterina S. Umnyakova, Nikolay P. Gorbunov, Alexander V. Zhakhov, Ilia A. Krenev, Tatiana V. Ovchinnikova, Vladimir N. Kokryakov, Mikhail N. Berlov

**Affiliations:** 1 Institute of Experimental Medicine, Acad. Pavlov Str. 12, Saint Petersburg 197376, Russia; umka-biolog@mail.ru (E.S.U.); kokryak@yandex.ru (V.N.K.); 2 Research Institute of Highly Pure Biopreparations, Pudozhskaya Str., 7, Saint Petersburg 197110, Russia; n.p.gorbynov@hpb.spb.ru (N.P.G.); a.v.zachov@hpb.spb.ru (A.V.Z.); 3 Department of Biochemistry, Saint-Petersburg State University, Universitetskaya Embankment, 7/9, Saint-Petersburg 199034, Russia; il.krenevv13@yandex.ru; 4 M.M. Shemyakin and Yu. A. Ovchinnikov Institute of Bioorganic Chemistry, Russian Academy of Sciences, Miklukho-Maklaya Str., 16/10, Moscow 117997, Russia; ovch@ibch.ru

**Keywords:** *Arenicola marina*, antimicrobial peptides, arenicin, complement, C3a

## Abstract

Antimicrobial peptides from marine invertebrates are known not only to act like cytotoxic agents, but they also can display some additional activities in mammalian organisms. In particular, these peptides can modulate the complement system as was described for tachyplesin, a peptide from the horseshoe crab. In this work, we investigated the influence on complement activation of the antimicrobial peptide arenicin-1 from the marine polychaete *Arenicola marina*. To study effects of arenicin on complement activation in human blood serum, we used hemolytic assays of two types, with antibody sensitized sheep erythrocytes and rabbit erythrocytes. Complement activation was also assessed, by the level of C3a production that was measured by ELISA. We found that the effect of arenicin depends on its concentration. At relatively low concentrations the peptide stimulates complement activation and lysis of target erythrocytes, whereas at higher concentrations arenicin acts as a complement inhibitor. A hypothetical mechanism of peptide action is proposed, suggesting its interaction with two complement proteins, C1q and C3. The results lead to the possibility of the development of new approaches for therapy of diseases connected with complement dysregulation, using peptide regulators derived from natural antimicrobial peptides of invertebrates.

## 1. Introduction

The biologically active compounds derived from marine organisms are known to be unique, and can be used as a pattern for the development of new, effective pharmacological agents for treatment of different diseases [1]. Antimicrobial peptides named arenicins are found in coelomocytes of the marine polychaete *Arenicola marina*. Three isoforms of arenicins have been described: arenicin-1, -2 [2] and -3 [3]. These cationic peptides consist of 21 amino acid residues, and have the structure of a β-hairpin [4,5] stabilized by one (for arenicins-1 and -2) or two (for arenicin-3) intramolecular disulfide bonds. Arenicins have also been detected in epithelia of the body wall and gut of *A. marina* [6]. At micromolar concentrations, these peptides demonstrate significant antimicrobial activity towards a wide range of Gram-positive and Gram-negative bacteria, as well as of fungi [2,3,7,8,9], and they seem to play a significant role in host defense of this polychaete. The mechanism of antimicrobial activity is realized by the action of peptide molecules on the microbial cytoplasmic membrane forming transmembrane pores [7,9,10]. Several attempts have been made to design a structure of peptide antibiotics derived from arenicins [11,12,13,14]. As potential medical drug prototypes, arenicins should be tested for their biological effects beyond direct antimicrobial activity.

**Figure 1:**
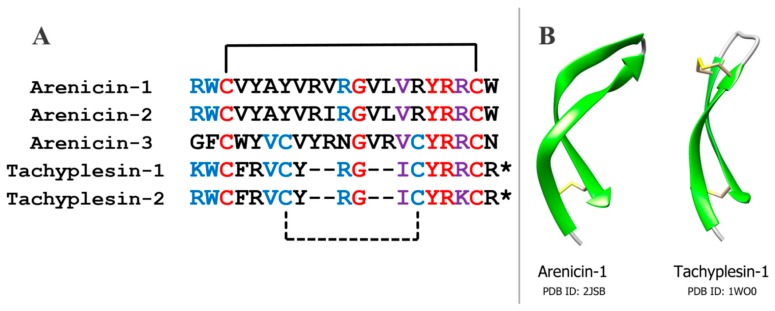
Structural similarities between arenicins and tachyplesins. (**A**) Multiple sequence alignment of three arenicins and two tachyplesins. Identical and highly similar residues in the same positions for all five peptides are colored with red and violet, respectively. Identical or highly similar residues shared by one or two arenicins and two tachyplesins are colored with blue. Black lines indicate cysteine pairing, the disulfide bond absent in arenicins-1 and -2 shown as a dashed line. Asterisks indicate amidated arginine residues. (**B**) Spatial structures of arenicins-1 and tachyplesin-1; images were generated with Chimera 1.11 software.

Arenicins have similar amino acid sequence and spatial structure to another group of antimicrobial peptides, tachyplesins, which have been isolated earlier from the horseshoe crab *Tachypleus trindentatus,* and polyphemusins from *Limulus polyphemus*, which are related to tachyplesins [15,16] (Figure 1). Tachyplesins, like arenicins, have a β-hairpin structure that is stabilized by two intramolecular disulfide bonds. This similarity indicates that these peptides may belong to a common family of antimicrobial peptides [17]. On the other hand, the structures of arenicins’ and tachyplesins’ precursors, and their posttranslational processing, are quite different [2,18]. Thus, the evolutionary relationship between these peptides remains questionable.

It has been shown that tachyplesin-1 is capable of forming a complex with human C1q complement protein that leads to antibody-independent complement classical pathway activation in human blood serum. In particular, tachyplesin can bind to the surface of TSU human prostate carcinoma cells, making these cells a target for complement action [19]. It was determined that the interaction of tachyplesin with C1q requires the integrity of peptide spatial structure, since the reduction and alkylation of disulfide bonds lead to a weaker binding to C1q.

The complement system is a part of the immune defense in mammals, represented by a network of serum proteins (complement components) [20,21]. Complement activation leads to opsonization of target cells, or their lysis by the membrane attack complex (MAC). The latter event is usually restricted to Gram-negative bacteria, however host cells, especially erythrocytes, may also be lysed by MAC. There are three main pathways of complement activation, named the classical, alternative, and lectin pathways. All of them converge on proteolytic cleavage of C3 component to C3a and C3b by so-called C3-convertases. After the cleavage, C3b is able to bind covalently to hydroxyl-containing molecules via its intrinsic thioester bond. This can mediate its accumulation on the surface of target cells, normally microbes or apoptotic cells. C3b is incorporated into C3-convertases, changing their specificity for C5 component cleavage, which initiates MAC assembly.

According to the structural similarity between arenicins and tachyplesins, we assumed that arenicin could also interact with C1q and that this might influence the complement activation. In a previous paper, we demonstrated that arenicin-1 really forms a stable complex with human C1q [22].

In this paper, we studied the effects of arenicin-1 on complement activation and target cell lysis in two hemolytic models in vitro. We also used an ELISA test for C3a to confirm the complement activation in these models. We found that the effect of arenicin on the complement system depends on the concentration of the peptide. At relatively low concentrations, arenicin stimulates complement activation and/or target erythrocyte lysis. However, at higher concentrations arenicin behaves as a complement system inhibitor. Though it was not demonstrated directly, our results provide strong evidence about the interaction of arenicin with the C3 complement protein, in addition to the previously established interaction with C1q.

## 2. Results

**Figure 2:**
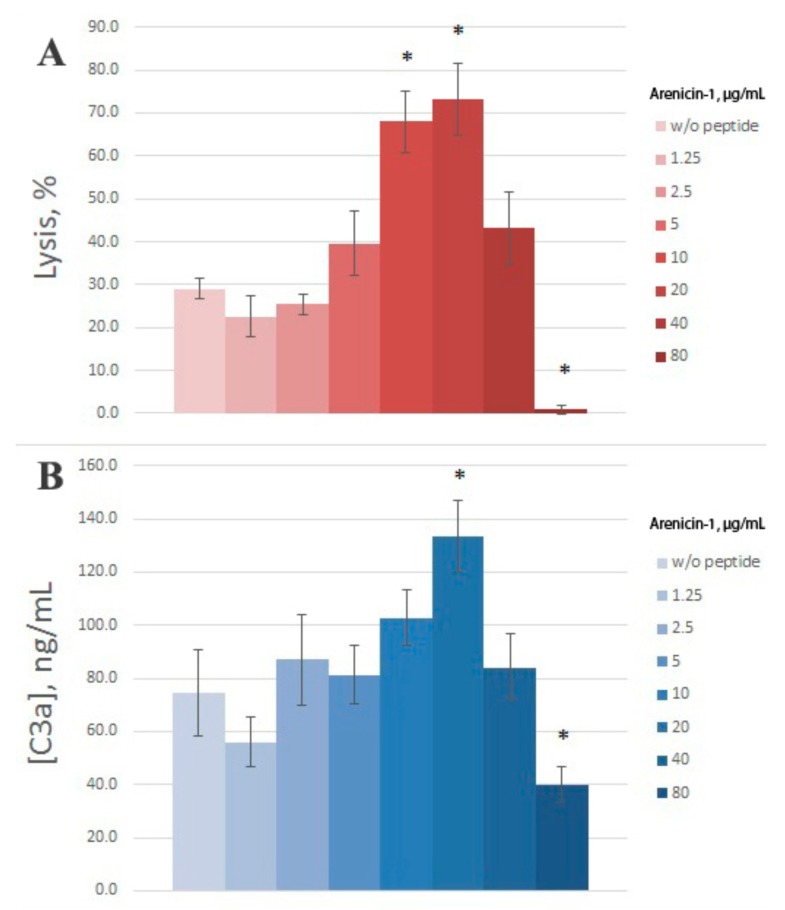
The action of arenicin-1 on complement activation and lysis of antibody sensitized sheep erythrocytes (E^sh^). Data are represented as mean ± SD (*n* = 5). * *p* < 0.05 vs. control (samples without peptide). (**A**) E^sh^ lysis level, %; (**B**) C3a concentration in samples, ng/mL.

In the hemolytic assay with antibody sensitized sheep erythrocytes (E^sh^), which is the model for the classical pathway of complement activation, we observed more than twice the baseline level of E^sh^ lysis by 1% normal human serum (NHS) in the presence of arenicin-1 at concentrations 10 and 20 μg/mL, compared with control (samples without peptide) (Figure 2A). However, at higher concentrations (80 μg/mL), arenicin displayed a reverse effect and almost totally abolished complement-mediated hemolysis. In all concentrations tested, arenicin itself revealed no hemolytic activity in experimental conditions, because in samples with active serum replaced for heat-inactivated serum there was no lysis above background level (data not shown). 

To assess whether E^sh^ lysis level differences reflect different complement activation levels, we developed an ELISA system for human C3a, a derivative of the complement protein C3, which is produced on complement activation. The test system was suitable for detection of C3a in the range 25–500 ng/mL, with no cross-reactivity to the uncleaved C3 protein.

**Figure 3:**
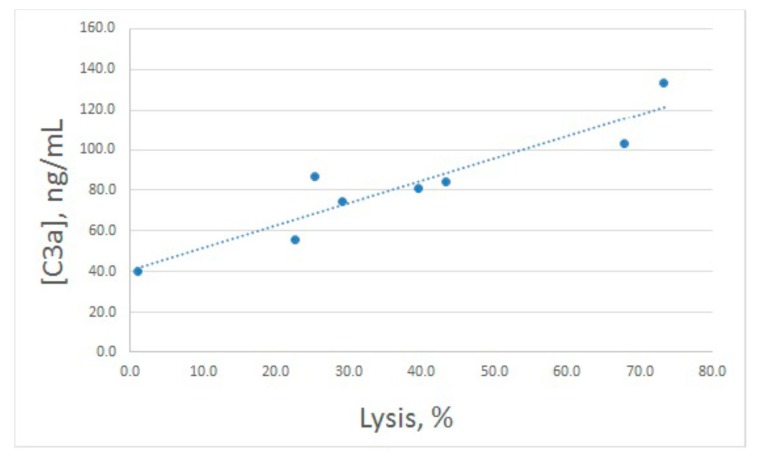
Correlation between percentages of antibody sensitized sheep erythrocytes lysed by human serum at different concentrations of arenicin-1 and C3a production in these samples. Pearson correlation coefficient was calculated as 0.93.

After hemolytic assay, the same samples were used for C3a determination (Figure 2B). Both increased and decreased lysis levels were accompanied by concerted alterations in C3a production, though these alterations were less prominent compared with lysis. In fact, the enhanced value of C3a differed significantly from the control one only for 20 μg/mL of arenicin. The relationship between E^sh^ lysis and C3a production is illustrated in Figure 3. Pearson correlation coefficient was calculated as 0.93, which is significant for *p* < 0.05.

**Figure 4:**
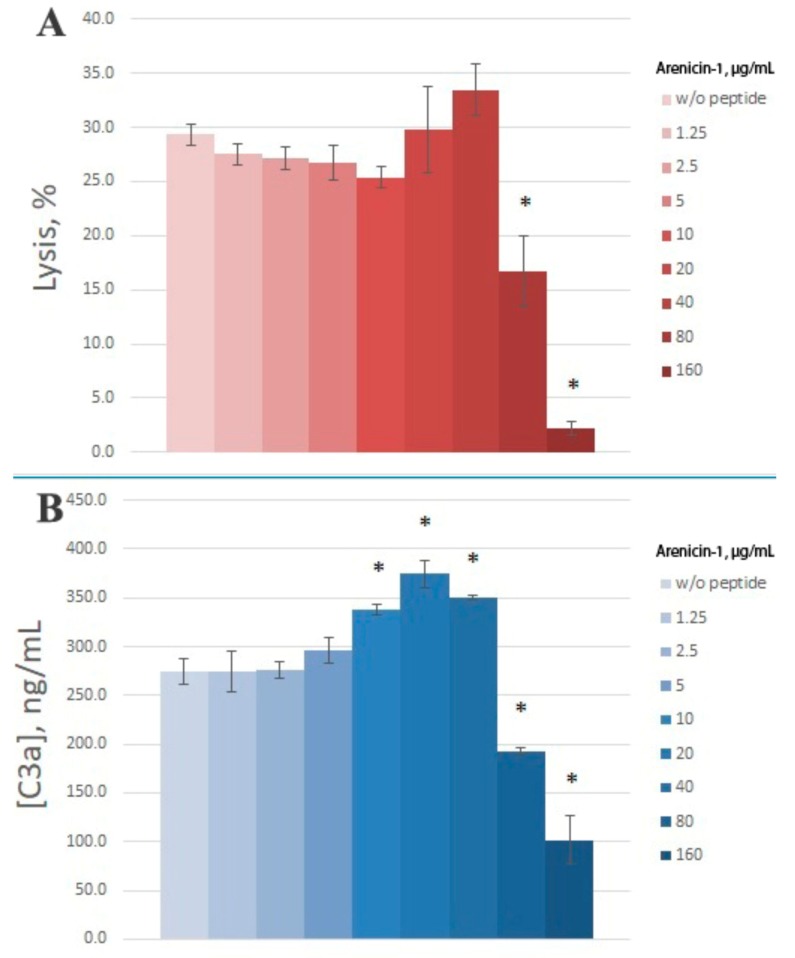
The action of arenicin-1 on complement activation and lysis of rabbit erythrocytes (E^rab^). Data are represented as mean ± SD (*n* = 5). * *p* < 0.05 vs. control (samples without peptide). (**A**) E^rab^ lysis level, %; (**B**) C3a concentration in samples, ng/mL.

As a model of complement activation via the alternative pathway, a hemolytic assay with rabbit erythrocytes (E^rab^) is widely used. We utilized this assay to study arenicin action on the complement system as well. Since we used 4% serum in this model, we took a broader concentration range of arenicin. We found that in this model, arenicin retained its inhibitory action at high concentrations (80 and 160 μg/mL), essentially decreasing hemolysis level (Figure 4A). No significant effect was observed for lower concentrations of the peptide. Similar to the previous model, arenicin did not induce E^rab^ lysis itself in heat-inactivated serum (data not shown).

**Figure 5:**
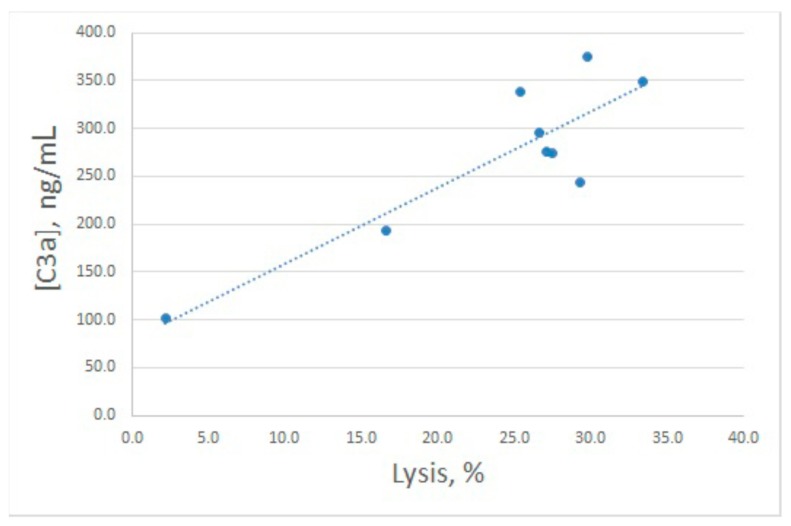
Correlation between percentages of rabbit erythrocytes lysed by human serum at different concentrations of arenicin-1 and C3a production in these samples. Pearson correlation coefficient was calculated as 0.88.

Inhibitory action of arenicin on E^rab^ lysis by NHS was confirmed to reflect its influence on complement activation, since C3a levels in the samples with high arenicin concentrations (80 and 160 μg/mL) were significantly diminished (Figure 4B). Unexpectedly, we found that in the presence of arenicin at 10–40 μg/mL, a modest but reproducible increase in C3a production was observed in the E^rab^ model. While this did not lead to enhanced E^rab^ lysis, the Pearson correlation coefficient for this model was 0.88, which is significant for *p* < 0.05. Correlation between E^rab^ lysis and C3a generation is shown in Figure 5.

To sum up, complement activation stimulated by different types of erythrocytes was of an approximately equal level. Differences between C3a concentrations for control samples (Figure 2B and Figure 4B) are due to different serum dilution in these models (1:100 for E^sh^ and 1:25 for E^rab^). In both models, arenicin revealed both activating and inhibitory effects depending on its concentration.

## 3. Discussion

Previously, it has been demonstrated that the antimicrobial peptide tachyplesin interacts with C1q protein and activates the classical complement pathway. Generally, the classical pathway is initiated as a consequence of recognition by C1q of IgGs or IgMs in complex with their antigens. However, tachyplesin seems to activate the complement system independently of antibodies [19]. Since arenicin structurally resembles tachyplesin and interacts with C1q [22], a similar action of this peptide on the complement system was expected.

It is commonly accepted that antibody sensitized E^sh^ stimulates the classical pathway of the complement system and activation via the alternative pathway is insignificant, whereas E^rab^ hemolysis is a model for alternative pathway investigations. However, we observed a stimulating action of arenicin-1 in both hemolytic models, though it was expressed mainly in lysis level in the case of E^sh^, and in C3a production in the case of E^rab^. Unexpectedly, at high doses (80–160 µg/mL) arenicin displayed reverse effects, essentially diminishing lysis level and C3a generation, in both experimental systems. Apparently, high doses of arenicin inhibit both the classical and alternative pathways, and the latter cannot be explained by the interaction of arenicin with C1q. Instead, the inhibitory effect of arenicin must be related to the action on the common point of both pathways, i.e., C3 cleavage. Most likely, this is the interaction of arenicin with C3 protein leading to its protection from cleavage, although interactions of arenicin with C3-convertases are also possible.

There are three types of C3-convertases: C4b2a, a common C3-convertase of classical and lectin pathways; C3(H_2_O)Bb generated at spontaneous ‘tick-over’ activation of the complement cascade by the alternative pathway; C3bBb, a convertase of the ‘amplification loop’ of the complement system, also usually considered as part of the alternative pathway. The tick-over C3-convertase is an exclusively fluid phase enzyme, while the other two could be covalently bound to the surface of a target cell [23]. Our results imply that arenicin-1 is able to inhibit C3 cleavage by all these convertases.

It is more difficult to explain stimulating effects of arenicin on the complement system at relatively low concentrations. It could be assumed that interaction of arenicin with C1q can lead both to antibody-independent classical pathway activation (as it was described for tachyplesin [19]) and to accelerated antibody-dependent complement activation. These two types of action could be reflected in experiments with E^rab^ (not treated with antibodies) and sensitized E^sh^, respectively. In the case of using E^sh^, arenicin interacts with C1q near the cell surface because C1q is bound to antibodies to surface antigens. On the other hand, C1q remains in the fluid phase in the E^rab^ model, and most C3b generated by classical pathway C3-convertase will not be attached to the erythrocyte surface. Thus, both classical pathway and amplification loop C3-convertases locate mainly in a fluid phase where they are highly unstable [21,23], and do not turn to C5-convertases. This could be a reason why there is no visible effect on lysis. However, this interpretation is in contradiction with use of Ca^2+^-free buffer for E^rab^ hemolysis, which is not compatible with the classical pathway activation. Thus, stimulating effects of arenicin on the complement system remain enigmatic, at least for E^rab^ hemolysis assay.

While the antimicrobial peptide tachyplesin-1 was shown to activate the classical complement pathway [19], we described here that structurally similar arenicin-1 is able to activate or inhibit the complement system at different doses in two experimental models, corresponding to classical and alternative pathways of complement system activation. It is not clear whether these differences in mode of action reflect structural differences of the two peptides, or if they could be explained by different assay types. Further experiments are required to compare the effects of arenicin and tachyplesin directly.

The ability of arenicin-1 to act as a potent complement system inhibitor makes it a prospective molecular source for the design of novel therapeutics directed to the complement system. Currently, the almost complete lack of therapeutic agents that regulate the level of complement activation is a serious medical problem. When hyperactivated, the complement system is perhaps the most dangerous pro-inflammatory and cytotoxic machinery in the human body. The complement system plays a significant role in the pathogenesis of a number of diseases, including age-related macular degeneration, paroxysmal nocturnal hemoglobinuria, hereditary angioedema, and some kidney diseases such as atypical hemolytic uremic syndrome and membranoproliferative glomerulonephritis type II [24,25,26,27]. In addition, the complement system and its components are involved in the development and course of autoimmune pathological processes such as systemic lupus erythematosus, rheumatoid arthritis, autoimmune hemolytic anemia; neurodegenerative and neoplastic diseases; and also complications after heart attacks, strokes and transplantations. Existing therapy for diseases connected with complement dysregulation is very expensive, and unavailable for the majority of patients.

Our results indicate that at low concentrations arenicin-1 acts differently on the complement system, depending on the presence of antibodies detecting target cells. Although we described this action as complement activation in both cases, fluid phase C3 conversion could be regarded as a kind of complement inhibition, since the C3 pool becomes depleted (C3 consumption). However, the host cells could also be targets of the complement system, for example in the case of autoimmune disorders. Moreover, C3a, a moderate anaphylatoxin, is produced due to fluid phase C3 conversion, which can induce some proinflammatory effects [28,29]. If one would like to escape these side-effects and obtain a “pure” complement inhibitor based on arenicin, there is a need for an arenicin derivative devoid of its stimulating activity on C3 conversion at low doses, but retaining the ability to protect C3 from cleavage at higher doses.

Arenicin-1 is known as a hemolytic peptide for human erythrocytes [8]. It could be expected to be a complication when using hemolytic assays for studying arenicin action on the complement system. However, as it was described above, no direct hemolytic action of arenicin was observed in our experiments. As we observed in human erythrocytes, supplementing buffer with gelatin is enough to reduce significantly the hemolytic activity of arenicin-1 (unpublished data). Presumably, gelatin presence is also the reason for the resistance of sheep and rabbit erythrocytes to arenicin in our experiments. Alternatively, serum components could protect erythrocytes from the lytic action of arenicin. It cannot be excluded that complement proteins are those serum components inhibiting the hemolytic action of arenicin, and thus, the complement system in turn can modulate the activity of arenicin. In any case, we cannot exclude the interaction of arenicin with erythrocyte membrane, which can influence the rate of erythrocyte lysis by MAC.

In conclusion, we revealed that arenicin-1 is able to both activate and inhibit the complement system in utilized test models in vitro. Our results imply that arenicin can be considered as the basis for the development of a new therapeutic drug for complement system modulation. In addition, the ability of arenicin to interact with the complement system should be taken into account in attempts to create novel antibiotic drugs derived from its structure. The presented observations should be treated as initial results due to the application of relatively simple methods of analysis of the complement system activation. The assays were conducted only with diluted serum, which is not physiologically relevant. In vivo tests on an animal model were not conducted. Further investigations are required to clarify mechanisms of arenicin action on the complement system, and its feasibility under in vivo conditions.

## 4. Materials and Methods 

### 4.1. Peptides

Arenicin-1 was synthesized based on the standard 9-fluorenylmethoxycarbonyl (Fmoc) protocol with the *O*-(benzotriazol-1-yl)-*N*,*N*,*N*′,*N*′-tetramethyluronium tetrafluoroborate/*N*,*N*-diisopropylethylamine (TBTU/DIEA) activation, using Wang-resin as the solid-phase and triphenylmethyl protecting groups for cysteines, as described previously [7]. Human C3a was kindly provided by Dr. A.M. Ischenko (Research Institute of Highly Pure Biopreparations, Saint Petersburg, Russia).

### 4.2. Serum and Erythrocytes

Normal human serum (NHS) was collected by medical staff (Laboratory of Viral Infections Diagnostics, Department of Clinical Microbiology, Pavlov First Saint Petersburg State Medical University) from more than 20 healthy volunteers, pooled, aliquoted, and stored at −70 °C no longer than two months. Serum aliquots were thawed at 4 °C in the day of the experiment, kept in ice baths before introducing to test tubes, and were not used repetitively. To obtain serum with an inactivated complement system, it was incubated at 56 °C for an hour immediately before the experiment.

Animal erythrocytes were purified from whole blood of rabbit and sheep. They were stored in Alsever’s solution at 4 °C for no more than 5 days. Before use, they were washed with an appropriate buffer: DGVB^++^ (dextrose gelatin veronal buffer with Ca^2+^ and Mg^2+^) for sheep erythrocytes (E^sh^), and GVB^+^ (gelatin veronal buffer with Mg^2+^) for rabbit erythrocytes (E^rab^). DGVB^++^ is a 2.5 mM sodium barbital buffer containing 71 mM NaCl, 150 mM glucose, 1 mM MgCl_2_, 0.15 mM CaCl_2_, 0.05% gelatin; pH 7.35. GVB^+^ is a 2.5 mM sodium barbital buffer containing 150 mM NaCl, 10 mM Mg-EGTA, 0.05% gelatin; pH 7.35. Before experiments, sheep erythrocytes were sensitized with antibodies (anti-sheep red blood cell stroma antibodies produced in rabbits, S1389, Sigma, St. Louis, MO, USA); we used a 1:1600 dilution of these antibodies and incubated sheep red blood cells for 30 min at 37 °C.

### 4.3. Hemolytic Assays

Hemolytic functional assays were performed utilizing erythrocytes (E^sh^ and E^rab^) as target cells as described elsewhere [30], with some modifications. Sensitized sheep erythrocytes were used for evaluation of complement activation via the classical pathway; to measure the activity of alternative pathway, rabbit erythrocytes were used. 

Experimental samples contained erythrocytes, diluted NHS as a source of complement proteins, and arenicin at different concentrations. For the classical pathway assay, E^sh^ were introduced to a final concentration of 5 × 10^8^ cells per mL, serum was diluted to 1%, and DGVB^++^ was used to dilute all the components. For the alternative pathway assay, there were 2.5 × 10^8^ cells per mL of E^rab^, 4% NHS, and GVB^+^. Some samples contained no serum and were used further as a blank. Inactivated serum was also used to control the effect of arenicin on erythrocytes in the absence of an active complement system. 

The incubation was carried out for half an hour at 37 °C. After the incubation, the lysis of the erythrocytes was stopped by the addition of PBS (phosphate buffered saline, pH 7.4) in a ratio of 1:7.5. To some test tubes, distilled water was added instead of buffer, and thus samples were obtained with 100% lysis. Samples were centrifuged at 500× *g* for 5 min at room temperature, and then hemoglobin was determined in supernatants by measuring the optical density at 414 nm (OD_414_) in a microplate reader (Multiskan MS, Labsystems, Vantaa, Finland). After measurement, the samples were used for C3a determination by ELISA.

### 4.4. C3a Determination by ELISA

#### 4.4.1. Monoclonal Antibodies for C3a

For the immunization of F2-hybrid mice, 0.5 mg of C3a peptide in 1 mL of PBS was mixed with equal volume of Freund’s complete adjuvant (or Freund’s incomplete adjuvant for reimmunization). The reimmunization was carried out after 30 days; 12 days after that, the lymphocytes from the inguinal, peritoneal and axillary lymph nodes were collected for hybridization with Sp2/0 myeloma cells in the ratio 2:1. Cell hybridization was performed in PEG/DMSO (Sigma), using serial dilution with culture media [31]. The hybrids were transferred to 96-well culture plates, and cultivated for 12–16 days in RPMI-1640 medium (Sigma) containing 20% fetal calf serum. The HAT supplement (Sigma) was added to the culture medium solution for selection of hybridomas.

Clone screening was performed using ELISA in a 96-well microplate. Briefly, 50 µL of culture media was added to wells precoated with C3a antigen, and after incubation for 1 h, peroxidase-conjugated goat anti-mouse IgGs were added. Detection was performed with 3,3′,5,5′-tetramethylbenzidine (TMB) substrate solution.

For C3a ELISA development, two monoclonal murine antibodies to C3a, with different epitope specificities designated as CC3a-5 and CC3a-1, were selected. CC3a-5 was immobilized on a solid phase as a capture antibody, while CC3a-1 was conjugated with horseradish peroxidase and was used as a detection antibody.

#### 4.4.2. ELISA System for C3a

ELISA for C3a was performed in 96-well microplates. Wells of a microplate were coated with capture antibodies and blocked by 1% BSA in PBS, pH 7.2. The same BSA solution was used as diluent on subsequent steps. Ten-fold diluted experimental samples and calibration samples were introduced to the wells, after which the plates were incubated for 1 h at 37 °C. Then, 100 µL of peroxidase-conjugated detection antibodies were added to each well. After incubation for 1 h at 37 °C, the TMB substrate solution (Xema Co. Ltd., Moscow, Russia) was added. After 15–20 min, the reaction was stopped by adding sulfuric acid, and the absorbance at 450 nm was measured in a microplate reader (Multiscan MS, Labsystems).

### 4.5. Statistical Analysis

Statistical significance of the values was evaluated by paired *t*-test using the software package STATISTICA (version 7.0, TIBCO Software Inc., Palo Alto, CA, USA). All of the experiments were repeated five times. All data are presented as mean ± standard deviation (SD). Pearson coefficients were calculated and used for evaluation of the correlation level between C3a concentration and percentage of lysed cells. For both tests, *p*-values of less than 0.05 were considered statistically significant.
